# Prevalence, Virulence Characterization, AMR Pattern and Genetic Relatedness of *Vibrio parahaemolyticus* Isolates From Retail Seafood of Kerala, India

**DOI:** 10.3389/fmicb.2020.00592

**Published:** 2020-04-07

**Authors:** Sreejith V. Narayanan, Toms C. Joseph, Shaheer Peeralil, Mukteswar P. Mothadaka, Kuttanappilly V. Lalitha

**Affiliations:** ^1^Microbiology Fermentation and Biotechnology Division, ICAR-Central Institute of Fisheries Technology, Kochi, India; ^2^Cochin University of Science and Technology, Kochi, India

**Keywords:** pathogen, seafood, *V. parahaemolyticus*, *tdh*, PFGE, pandemic, antimicrobial resistance

## Abstract

*Vibrio parahaemolyticus*, a halophilic bacterium often found in the marine or estuarine environment is a well-known enteropathogen responsible for foodborne outbreaks associated with seafood. The pathogenic strains of *V. parahaemolyticus* are marked by the presence of thermostable direct hemoylsin (*tdh*) and/or TDH related hemolysin (*trh*) genes. This study aimed to investigate the prevalence and characteristics of potentially pathogenic *V. parahaemolyticus* in selected retail markets of Cochin, Kerala, along the south-western coast of the Indian subcontinent. One hundred samples collected from 10 retail markets were analyzed for the presence of pathogenic isolates of *V. parahaemolyticus*. Out of the 721 presumptive isolates, 648 were confirmed to be *V. parahaemolyticus* by *toxR* gene amplification, among which 29 were Kanagawa phenomenon (KP) positive. Among these potentially pathogenic isolates, 17 possessed the *tdh* gene whereas none of them had the *trh* gene. The faint amplification bands produced during the amplification of *tdh* gene from two isolates was confirmed by sequencing. Multiplex O serotyping identified O1 serotype as the most prevalent serotype among the 29 potentially pathogenic isolates. Further, studies on the pandemic nature of these isolates revealed that 14 of the 29 were positive for the PGS-PCR, whereas all the isolates were negative for GS-PCR and HU-α PCR. The antibiogram of the isolates revealed that three isolates had significant Multiple Antibiotic Resistance (MAR) index of 0.2 or above. Pathogenic isolates resistant to second, third and fourth generation Cephalosporins were found to be present in the seafood studied. The molecular fingerprinting studies using ERIC-PCR, and PFGE revealed that three of these isolates shared close genetic similarities with the clinical strains. The environmental and seafood isolates that produced faint amplification bands during the amplification of *tdh* gene suggests that the *tdh* gene often goes undetected in environmental isolates. The conventional methods used to identify the pathogenic *V. parahaemolyticus* would be good for clinical isolates, but a more elaborate method is recommended for the detection of *tdh* gene in environmental isolates. This is the first comprehensive study on pathogenic *V. parahaemolyticus* in Kerala, India and demonstrates for the first time, the isolation of potentially pathogenic *V. parahaemolyticus*, carrying *tdh* gene from seafood collected from retail markets in Kerala.

## Introduction

*Vibrio parahaemolyticus* is a Gram-negative, halophilic bacterium often associated with seafood borne gastrointestinal illness. Ever since the discovery of *V. parahaemolyticus* (Fujino et al., 1953) from Shirasu associated outbreak in Japan, the organism has been found to be responsible for seafood-borne illness worldwide (Nair et al., 2007). In Asia, *V. parahaemolyticus* related foodborne outbreaks have been reported in many countries including Japan, India, China, Taiwan, Bangladesh, and Malaysia (Matsumoto et al., 2000; Letchumanan et al., 2014). In India, *V. parahaemolyticus* was first isolated from a case of gastroenteritis by Chatterjee et al. (1970). Later, *V. parahaemolyticus* belonging to serotypes O4:K12 and O1:K20 was found to cause a diarrheal outbreak in Vellore, Tamil Nadu, in 1982 (Lalitha et al., 1983).

The pathogenic isolates of *V. parahaemolyticus* were differentiated from non-pathogenic isolates on the basis of “Kanagawa phenomenon” (KP), the ability of the bacterium to lyse human erythrocytes, that is linked with the production of a pore-forming toxin called the thermostable direct hemolysin (TDH) (Miyamoto et al., 1969; Honda et al., 1992; Wang et al., 2015). Although many virulence factors viz. ToxRS, siderophores, type III secretory system, Type VI secretion system has been described in *V. parahaemolyticus*, the major virulence factors remains to be TDH and TDH related hemolysin (TRH) which are often used as markers for detecting pathogenic *V. parahaemolyticus* (Tada et al., 1992). The dramatic increase in the incidence of *V. parahaemolyticus* infections and epidemics since 1996, was attributed to the emergence of a pandemic clone, belonging to the O3:K6 serovar (Nair et al., 2007). Identical strains were reported from Taiwan, Laos, Japan, Korea and the United States (Matsumoto et al., 2000). The potential of this pandemic clone to cause an outbreak created renewed interest among the researchers and over the time several PCR based assays such as GS-PCR, PGS-PCR, HU-α PCR were developed for the rapid detection of pandemic clones (Matsumoto et al., 2000; Okura et al., 2004; Williams et al., 2004).

Molecular subtyping is important in epidemiological investigations for recognizing outbreaks, detecting cross contamination, determining the source of infection, identifying virulent strains, and monitoring vaccinations programs (Olive and Bean, 1999). DNA based subtyping methods also facilitate the identification of pandemic strains (Chowdhury et al., 2000). Several molecular methods have been developed for the subspecies typing of *V. parahaemolyticus*, namely ERIC-PCR, Ribotyping, PFGE, REP-PCR and lately MLST (Matsumoto et al., 2000; Wong and Lin, 2001; González-Escalona et al., 2008).

The recent emergence and spread of antibiotic resistance reported among *V. parahaemolyticus* (Li et al., 2014; Letchumanan et al., 2015; Ahmed et al., 2018) is certainly the most striking evolution that has arisen in this pathogenic bacteria. The emergence of multidrug-resistant phenotypes among *V. parahaemolyticus* in many countries is an increasing concern (Letchumanan et al., 2014; Elmahdi et al., 2016). Despite the rise in reports of *V. parahaemolyticus* food-borne infections worldwide and the alarming increase in the prevalence of multidrug-resistant *V. parahaemolyticus*, little information is available in India, on the prevalence and characteristics of pathogenic *V. parahaemolyticus* in retail markets (Sanjeev and Stephen, 1995; Dileep et al., 2003; Pal and Das, 2010; Parthasarathy et al., 2016). In the present study, we performed a comprehensive analysis of various attributes including the biochemical, virulence and genetic characteristics, serotypes, pandemicity, antimicrobial resistance profile, and DNA fingerprints of pathogenic *V. parahaemolyticus* isolated from the seafood frequently consumed by the population in Kerala (India). The multidrug resistant nature of the *V. parahaemolyticus* seafood isolates and the genetic relatedness of the seafood isolates to that of the pandemic strains were assessed in this study.

## Materials and Methods

### Sample Collection

One hundred samples comprising of fish and shellfish frequently consumed were collected from 10 retail markets in Cochin, Kerala, situated along the south-western part of the Indian subcontinent. The sampling sites were spread across an area of 80 square miles. Five samples each were drawn in two intervals from each of the 10 retail markets under study (Table 1). Sampling was avoided during the monsoon months (South west monsoon-June, July; North East monsoon- Mid-October to Mid-November). The samples collected consisted of 32 shellfish samples, 63 fish samples, and 5 water samples. The five water samples were taken from fish vendors from five retail markets who used this water to sprinkle over the fish to keep its freshness in the absence of ice. Of the 10 retail markets studied, one retail market was in a landing center. All the samples were collected under aseptic conditions, transported to the laboratory in iced containers and analyzed within 3 h of collection adhering to the standard biosafety norms [IBSC approval number: BT/BS/17/740/2017-PID dated 10th November 2017 (DBT India)].

**TABLE 1:** Sampling locations.

**Sl. No**	**Location**	**Coordinates**	**Period of sampling**
1	Thevara	9°56′38.8′′N 76°17′37.9′′E	January 2015 August 2015
2	Polakandam	9°56′21.1′′N 76°15′17.6′′E	January 2015 September 2015
3	Chempu	9°49′14.7′′N 76°23′24.6′′E	March 2015 January 2016
4	Aroor	9°52′46.0′′N 76°18′12.6′′E	April 2015 August 2016
5	Edavanakad	10°06′12.4′′N 76°12′15.1′′E	May 2015 March 2016
6	Varapuzha	10°04′42.7′′N 76°16′14.3′′E	May 2015 February 2016
7	Chandroor	9°50′28.8′′N 76°18′32.5′′E	September 2015 April 2016
8	Thoppumpady	9°55′50.9′′N 76°16′02.5′′E	November 2015 September 2016
9	Broadway	9°58′53.7′′N 76°16′39.6′′E	December 2015 August 2016
10	Palluruthy	9°55′11.7′′N 76°16′24.9′′E	December 2015 September 2016

### Isolation and Biochemical Characterization

For the enrichment and isolation of *V. parahaemolyticus* from seafood samples standard US FDA protocol was followed with minor modifications (Kaysner and DePaola, 2004). All the media used for the isolation and identification of *V. parahaemolyticus* contained 3% NaCl. Samples of muscle tissue (25 g) were aseptically weighed into 225 ml of Alkaline Peptone Water (APW) (pH 8.5–9.0) and homogenized for 30 s with a stomacher (Lab blender 400, Seward Ltd., United Kingdom) and incubated at 37°C for 18 h. After incubation, 1 ml from the enrichment broth was serially diluted up to 10^–4^ dilutions and 0.1 ml from 10^–2^, 10^–3^, and 10^–4^ dilutions were plated on to Thiosulphate Citrate Bile salts Sucrose (TCBS) Agar (BD, United States). From each sample ten characteristic *V. parahaemolyticus* colonies were picked onto Tryptic Soy Agar (TSA) (BD, United States) slants and purified. Biochemical tests such as Oxidase, Catalase, growth in 0%, 3%, 6%, 8%, and 10% NaCl, and O/129 susceptibility were employed for the tentative identification of the *Vibrio* species. The reference strain *V. parahaemolyticus* ATCC 17802 was used as positive control.

### Molecular Confirmation of *V. parahaemolyticus*

A total of 721 presumptive colonies of *V. parahaemolyticus* isolated from positive samples were subjected to *toxR* gene amplification using the protocol described by Kim et al. (1999). The primers and amplification protocol used are described in Table 2. Crude DNA lysate was used as the template for the PCR reactions. All PCR reactions were carried out in Veriti^TM^ 96-Well Thermal Cycler (Thermo Fisher Scientific, United States).

**TABLE 2:** Primers and amplification protocols.

*toxR*	5’-GTCTTCTGACGCAATCGTTG-3’ (forward) 5’-ATACGAGTGGTTGCTGTCATG-3’ (reverse)	368	Kim et al., 1999	95°C-30 s 63°C-30 s 72°C-30 s
*tdh*	5′-GTAAAGGTCTCTGACTTTTGGAC -3’ (forward) 5′-TGGAATAGAACCTTCATCTTCACC-3′ (reverse)	269	Bej et al., 1999	94°C-1 min 58°C-1 min 72°C-1 min
	5’-CTGTCCCTTTTCCTGCCCCCG-3’(forward) 5’-AGCCAGACACCGCTGCCATTG-3’(reverse)	245	Gutierrez West et al., 2013	95°C-1 min 62°C-1 min 72°C-1 min
*trh*	5’-GGCTCAAAATGGTTAAGCG-3′ (forward) 5’-CATTTCCGCTCTCATATGC-3’ (reverse)	250	Tada et al., 1992	95°C-1 min 55°C-1 min 72°C-1 min
PGS PCR	5′-TTCGTTTCGCGCCACAACT-3′ (forward) 5′-TGCGGTGATTATTCGCGTCT-3′ (reverse)	235	Okura et al., 2004	94°C-30 s 60°C-30 s 72°C-30 s
GS PCR	5’-TAATGAGGTAGAAACA-3’(forward) 5’-ACGTAACGGGCCTACA-3’ (reverse)	651	Matsumoto et al., 2000	96°C-1 min 45°C-2 min 72°C-3 min
HU-α	5′-CGATAACCTATGAGAAGGGAAACC-3′ (forward) 5′-TAGTAAGGAAGAATTGATTGTCAAATAATG-3’ (reverse)	474	Williams et al., 2004	95°C-30 s 56°C-30 s 72°C-30 s

### Detection of *V. parahaemolyticus* With Pathogenic Potential

#### Determination of Hemolytic Activity

Only *toxR* positive isolates (*n* = 648) were selected for testing the KP on Wagatsuma Agar as described by Miyamoto et al. (1969). Briefly, 5 ml of freshly drawn human blood was centrifuged at 5000 *g* for 5 min, the erythrocytes were washed thrice with normal saline and resuspended in normal saline to a final volume of 5 ml and added to basal Wagatsuma agar medium and plated. Overnight cultures grown on TSA slants were used to inoculate the plates. The inoculated plates were incubated at 37°C for 48 h, and isolates producing β-hemolysis were scored Kanagawa positive. The *V. parahaemolyticus* strain NICED.VP.459 (NICED, Kolkata, India) was used as the positive control.

#### Amplification of Virulence Genes

All the KP positive strains (*n* = 29) were subjected to *tdh* and *trh* gene amplification. To detect pandemic clones in KP positive strains, GS-PCR, PGS-PCR, and HU-α PCR were employed. Two sets of primers (Bej et al., 1999; Gutierrez West et al., 2013) were used for the amplification of *tdh* gene. The PCR amplification of genes was performed separately in 25 μl volume. The primers and amplification protocols used are described in Table 2. Crude DNA lysate was used as template for all the PCR reactions. *V. parahaemolyticus* O3:K6 reference isolate, *V. parahaemolyticus* ATCC 17802 and the *V. parahaemolyticus* clinical strains provided by the National Institute of Cholera and Enteric Diseases, Kolkata, NICED.VP458, NICED.VP459, NICED.VP460 were used as positive controls in various PCR amplifications.

For the amplification of *tdh* (Gutierrez West et al., 2013 primers), *trh* and the PGS-PCR, the reaction mixture contained 0.5 pmols/μl primer, 1.5 mM MgCl_2_ (Thermo Fisher Scientific, United States) and 1.25 units Taq DNA Polymerase (Thermo Fisher Scientific, United States). 0.2 pmols/μl primer, 1.5 mM MgCl_2_ (Thermo Fisher Scientific, United States) and 1.25 units Taq DNA Polymerase (Thermo Fisher Scientific, United States) was used for GS-PCR. 1 pmol/μl primer, 2 mM MgCl_2_, and 1.25 units Taq DNA Polymerase (Thermo Fisher Scientific, United States) was used for the amplification of *tdh* gene with the Bej et al. (1999) primers.

### Multiplex PCR for O Serotyping

PCR method described by Chen M. et al. (2012) was used for the identification of O serotypes of all KP positive isolates. Briefly, all isolates were subjected to PCR amplification using two sets of primers; PCR group 1 for the detection of the serogroups O1, O2, O4, O5, O6, and O10 and PCR group 2 for the detection of serogroups O3/O13, O7, O8, O9, O11, and O12. In each reaction of 25 μl, 2.5 mM MgCl_2_, 1.5 units Taq DNA polymerase (Thermo Fisher Scientific, United States) and primer concentration as described in the reference was used. The amplification conditions consisted of an initial denaturation at 95°C for 5 min, followed by 30 cycles of denaturation at 95°C for 30 s, annealing at 60°C for 45 s, extension at 72°C for 1 min, and a final extension at 72°C for 7 min.

### Cloning and Sequencing

The faint bands produced by two isolates during the amplification of *tdh* gene were sliced from the gel and gel elution performed using GenElute Gel Extraction Kit (Sigma-Aldrich, United States). The eluted product was used as template for the reamplification of the *tdh* gene using the same PCR protocol. The PCR product was cloned into the vector pTZ57R/T and transformed to *E. coli* JM109 using InsTAclone PCR Cloning Kit (Thermo Fisher Scientific, United States). The transformants were selected based on blue-white selection and the vector with insert was extracted from the transformants using GenElute Plasmid Miniprep Kit (Sigma-Aldrich, United States) and sequenced using the Sanger sequencing technology. BLAST algorithm was used for similarity searches in the Genbank Sequence Database.

### Antibiotic Resistance Profile

The antimicrobial susceptibility of 29 potentially pathogenic *V. parahaemolyticus* isolates from seafood and the ATCC 17802 reference strain was performed using the disk diffusion method in Mueller Hinton Agar (BD, United States) supplemented with 2% NaCl. Twelve antibiotics representing 8 classes were used in this study. The zone diameter was measured and the results were interpreted as Sensitive (S), Intermediate (I), and Resistant (R) based on Clinical and Laboratory Standards Institute (CLSI) breakpoints for *Vibrio* sp. with the help of WHONET software (O’Brien and Stelling, 1995). The antibiotics tested include Ampicillin (10 μg), Amoxycillin-Clavulanate (20/10 μg), Cefepime (30 μg), Cefotaxime (30 μg), Cefoxitin (30 μg), Ceftazidime (30 μg), Meropenem 10(μg), Gentamicin (10 μg), Tetracycline (30 μg), Ciprofloxacin (5 μg), Trimethoprim/Sulfamethoxazole (1.25/23.75 μg), and Chloramphenicol (30 μg). All the antibiotics discs used in this study were procured from Himedia, India. The Multiple Antibiotic Resistance (MAR) index for the isolates was calculated as described previously (Krumperman, 1983).

### Molecular Subtyping

#### ERIC-PCR

ERIC-PCR was performed using the protocol described by Wong and Lin (2001). Each reaction (50 ul) consists of 1 pmol/μl primers (ERIC1R, 5-ATGTAAGCTCCTGGGGATTCAC-3, and ERIC2, 5-AAGTAAGTGACTGGGGTGAGCG-3), 1.5 mM MgCl_2_ and 1.25 units DreamTaq DNA Polymerase (Thermo fisher Scientific, United States). The amplification conditions used for ERIC-PCR include an initial denaturation at 95°C for 5 min, denaturation at 95°C for 30 s, annealing at 52°C for 1 min, extension at 72°C for 5 min and a final extension at 72°C for 10 min. The PCR products were separated on a 2% agarose gel and imaged using G:Box Imaging System (Syngene).

#### PFGE

The standard operating procedure for PFGE of *V. cholerae* and *V. parahaemolyticus* from the Pulsenet was followed with minor modifications. Cell suspensions for making plugs were prepared from confluent growth on TSA plates using a sterile cotton swab. The OD of the cultures was adjusted between 0.9 and 1.0 in a spectrophotometer at 610 nm. The plugs were prepared in disposable plug molds after adding Proteinase K (Sigma-Aldrich, United States) to the cell suspension to a final concentration of 0.5 mg/ml. The plugs were subjected to lysis in cell lysis buffer in a shaker at 55°C for 1 h. The lysed plugs were washed twice with pre-warmed water and four times with pre-warmed TE Buffer. For the digestion of plug, 40 U of *Sfi*I (New England Biolabs, United States) was used for *V. parahaemolyticus* cultures and 50 U of *Xba*I (New England Biolabs, United States) was used for the digestion of *Salmonella* Braenderup H9812. The digested plugs were loaded in a 1% agarose gel (Bio-Rad Certified^TM^ Megabase Agarose, United States) and the fragments separated in Chef Mapper Pulsed Field Electrophoresis System (Bio-Rad, United States) for 19 h with an initial switch time of 10 s and final switch time of 35 s. The gels were stained with Ethidium Bromide and imaged using G:Box Imaging System (Syngene).

#### Analysis of Fingerprints

The analysis of fingerprints generated by ERIC-PCR and PFGE were performed in GelCompar II version (5.1) (Applied Maths NV, Belgium). The dendrogram representing the relationship between the isolates was created in the software using the Dice Similarity coefficient and cluster analysis by UPGMA (unweighted pair group method with arithmetic averages) method. Optimization and position tolerance value of 1.5% was used during the analysis of fingerprints (Kai et al., 2008). To distinguish the number of PFGE types, a Dice similarity coefficient cut-off of 90% was used (Shaaly et al., 2005).

## Results

### The Occurrence of *V. parahaemolyticus*

Out of 100 samples screened, 90 samples (90%) harbored *V. parahaemolyticus* and the organism was isolated from samples collected from all the 10 retail markets under study during both rounds of sampling. 721 bacterial isolates that exhibited similar biochemical reactions to that of *V. parahaemolyticus* for oxidase, catalase, salt tolerance and O129 sensitivity test were subjected to *toxR* gene amplification and 648 isolates that produced a band at 368 bp during electrophoresis were confirmed as *V. parahaemolyticus*. Presence of *V. parahaemolyticus* was confirmed in 31 out of 32 shellfish samples (96.8%), 56 out of 63 finfish samples (88.8%), and 3 out of 5 water samples (60%).

### Detection of *V. parahaemolyticus* With Pathogenic Potential

All the *toxR* positive isolates were subjected to hemolysis test on Wagatsuma agar. Among the 648 isolates, 29 isolates exhibited β-hemolytic activity (KP) and were subjected to further molecular characterization. These 29 potentially pathogenic *V. parahaemolyticus* strains were isolated from 11 samples (11%) including a water sample (Supplementary Table S1). Presence of potentially pathogenic *V. parahaemolyticus* was confirmed in 2 out of 32 shellfish samples (6.25%), 8 out of 63 finfish samples (12.7%), and 1 out of 5 water samples (20%). Out of 10 retail markets sampled, potentially pathogenic *V. parahaemolyticus* strains were isolated from samples collected from four retail markets.

#### Virulence Characterization

**FIGURE 1:**
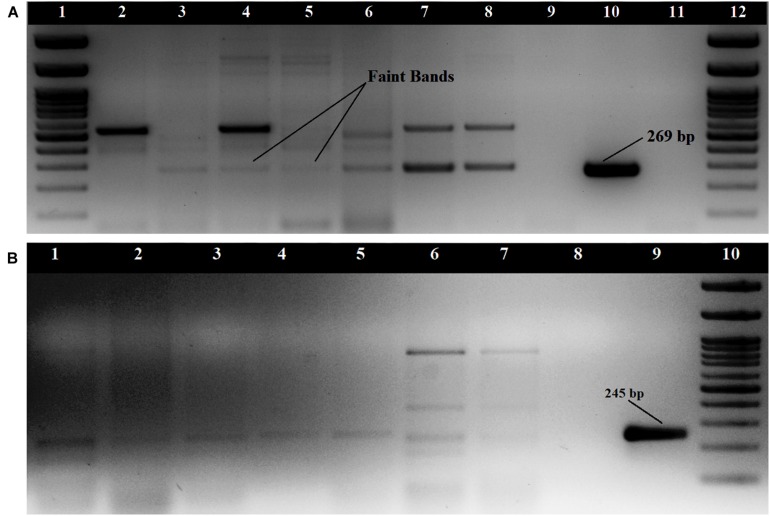
Amplification of *tdh* gene **(A)** with primers described by Bej et al. (1999); Loading order: **1.** 100 bp DNA Ladder **2.** RM.P.WT2 **3**. RM.P.WT3 **4**. RM.P.WT5 **5**.RM.P.PR2 **6**. RM.CH.CL5 **7.** RM.T.SH1 **8**. RM.T.SH6 **9**. ATCC 17802 **10**. NICED.VP459 **11**. Negative control **12**. 100 bp DNA Ladder **(B)** with primers described by Gutierrez West et al. (2013) Loading order: **1.** RM.T.SR8 **2.** RM.CH.CL5 **3.** RM.CH.KZ1 **4.** RM.A.MK1 **5.** RM.P.WT3 **6.** RM.T.SH7 **7.** RM.T.SH1 **8.** Negative control **9.** NICED.VP459(+) **10**. 100 bp DNA Ladder.

The *tdh* gene amplification of 29 KP positive isolates was done as singleplex PCR using two sets of primers. The primers by Bej et al. (1999) produced non-specific amplifications for some strains but showed amplification bands at 269 bp for 7 isolates and faint amplification bands for nine isolates (Figure 1A). The primers described by Gutierrez West et al. (2013) did not produce prominent amplification bands for any of the seafood isolates, but faint bands were observed for nine isolates at 245 bp (Figure 1B). Both the primers used produced prominent amplification bands for clinical strains used in this study. Altogether, 17 of the 29 KP positive isolates produced amplification for *tdh* gene. All the isolates were found to be negative for the *trh* gene. The 17 *tdh* positive *V. parahaemolyticus* strains were isolated from 7 (6.8%) samples including a water sample (Supplementary Table S1). The presence of pandemic clones of *V. parahaemolyticus* among the isolates was tested using PGS-PCR, GS-PCR, and HU-α PCR. Among the 29 isolates, 14 produced a band at 245 bp in PGS PCR and none of the isolates were positive for GS-PCR and HU-α PCR.

### Cloning and Sequencing

The faint amplification bands that were produced during *tdh* gene amplifications by two isolates (RM.CH.KZ3 and RM.P.WT5) were eluted, reamplified and the product cloned into the vector pTZ57R/T and sequenced using the M13 primers. The sequences showed 100% similarity with the *tdh* gene using the BLAST algorithm and were submitted to the NCBI sequence database with the following accession numbers; MK976027, MK953547.

### Multiplex O Serotyping

The multiplex O serotyping PCR performed for all isolates including the reference strains revealed that among the 29 potentially pathogenic isolates, the most prevalent serotype was O1 with 10 isolates, followed by O2 and O5 with five each, followed by O4 and O12 with three each followed by O10 with two and O3 with single isolate.

### Antibiotic Resistance Pattern

**FIGURE 2:**
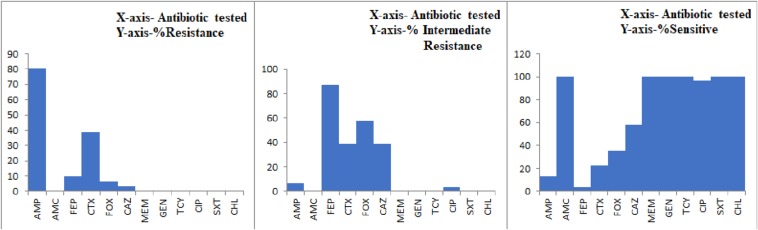
Graph representing the AMR profile of potentially pathogenic *V. parahaemolyticus* isolates from seafood.

All the 29 potentially pathogenic *V. parahaemolyticus* isolates were susceptible to Amoxycillin-Clavulanate, Gentamicin, Meropenem, Tetracycline, Trimethoprim/Sulfamethoxazole and Chloramphenicol (Table 3). Twenty-eight (96.6%) isolates were susceptible to Ciprofloxacin with one isolate (3.4%) showing intermediate sensitivity. Majority of the isolates were resistant to Ampicillin (79.3%). 41.4 and 10.3% of isolates were resistant to Cefotaxime and Cefepime, respectively (Figure 2). Multiple antibiotic resistances represented by MAR index of 0.2 or above were shown by two isolates from Mackerel (RM.A.MK1 and RM.T.MK1) and one isolate from Sardine (RM.T.SR12) (Supplementary Table S2).

**TABLE 3:** Interpretation of AMR profile of potentially pathogenic *V. parahaemolyticus* isolated from seafood (*n* = 29) as Sensitive (S), Intermediate (I), and Resistant (R) based on CLSI breakpoints.

**Antibiotic name**	**Antibiotic class**	**Code**	**Breakpoints**	**%R**	**%I**	**%S**
Amoxicillin/Clavulanic acid	Beta-lactam + Inhibitors	AMC	14–17	0	0	100
Ampicillin	Penicillins	AMP	14–16	79.3	6.9	13.8
Cefepime	Cephems	FEP	19–24	10.3	86.2	3.4
Cefotaxime	Cephems	CTX	23–25	41.4	34.5	24.1
Cefoxitin	Cephems	FOX	15–17	3.4	58.6	37.9
Ceftazidime	Cephems	CAZ	18–20	3.4	37.9	58.6
Chloramphenicol	Phenicols	CHL	13–17	0	0	100
Ciprofloxacin	Quinolones	CIP	16–20	0	3.4	96.6
Gentamicin	Aminoglycosides	GEN	13–14	0	0	100
Meropenem	Penems	MEM	20–22	0	0	100
Tetracycline	Tetracyclines	TCY	12–14	0	0	100
Trimethoprim/Sulfamethoxazole	Folate pathway inhibitors	SXT	11–15	0	0	100

### Molecular Subtyping

#### ERIC-PCR

**FIGURE 3:**
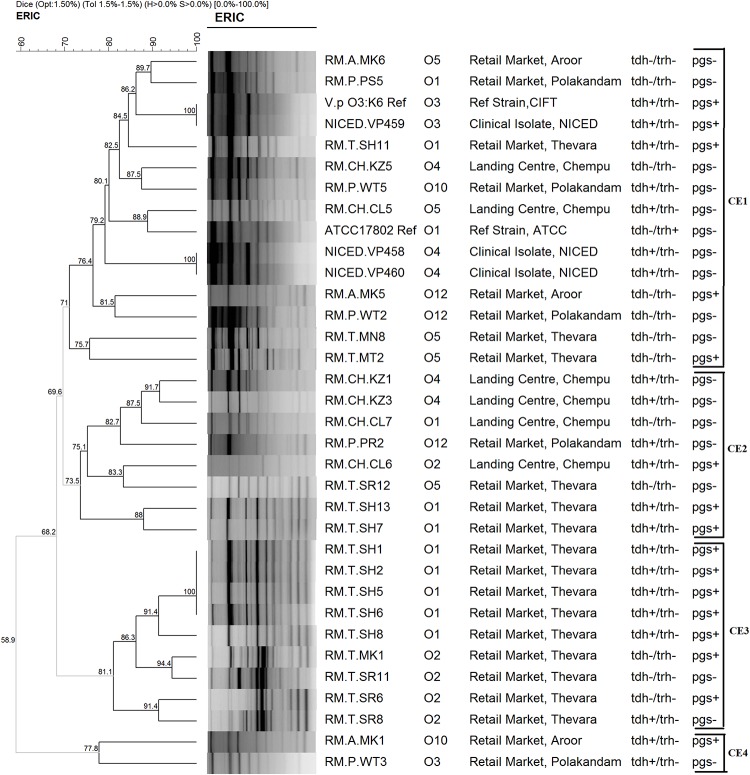
Dendrogram representing the cluster analysis using ERIC-PCR fingerprints.

All the isolates studied were typeable by ERIC-PCR. The fingerprints obtained using the ERIC2 primers had 9–14 bands ranging in size from 100 to 3000 bp. At a Dice similarity coefficient cut-off of 70%, the ERIC-PCR fingerprints distributed the isolates into four clusters (CE1, CE2, CE3, and CE4) among which the CE4 cluster had only two isolates (Figure 3). The minimum similarity among the isolates as known from the ERIC fingerprint analysis was 58.9%. Three among the four clusters (CE1, CE2, and CE4) had isolates from different geographical locations. The CE3 cluster had isolates only from a particular retail market (Thevara). CE1 formed the largest cluster with 15 isolates including all the clinical isolates and reference strains used in this study. Two isolates, RM.A.MK6 and RM.P.PS5, in CE2 showed 86.2% similarity with the pandemic strains used in this study. Grouping of isolates based on the virulence properties or O serotypes were not observed in the ERIC fingerprint based dendrogram.

#### PFGE

The separation of *Sfi*I digested genomic DNA in 1% agarose gel produced 14–19 fragments ranging in size from 20 to 670 kb. All the 29 isolates and the clinical isolates used in this study were typeable by PFGE. The analysis of fingerprint patterns using the Gel Compare II software grouped the isolates into six clusters (CP1, CP2, CP3, CP4, CP5, and CP6) at 70% Dice similarity coefficient cut-off and the minimum similarity among the isolates was found to be 64.5% (V). Two of the isolates, RM.T.SR11 and RM.CH.KZ3, didn’t cluster with other isolates at this cut-off. One isolate (RM.A.MK6) was found to cluster along with the non-pandemic clinical isolates NICED.VP458 and NICED.VP460 and the *V. parahaemolyticus* ATCC17802 strain in CP5. In cluster CP4, isolate RM.T.MN8 clustered along with the pandemic clinical isolates NICED.VP459 and *V. parahaemolyticus* O3:K6 Reference isolate. Clusters CP2 and CP3 had isolates from three different retail markets. Except for one isolate all isolates grouped in CP1 belonged to a single retail market. All clusters had the presence of isolates with different O serotypes. Thirteen distinct PFGE patterns were identified at a Dice similarity threshold of 90%.

## Discussion

In this study, potentially pathogenic *V. parahaemolyticus* was isolated from various seafood comprising of finfish and shellfish, and also from water samples. Presence of *V. parahaemolyticus* was confirmed in 90 of the 100 samples analyzed. A previous study on the prevalence of *V. parahaemolyticus* in fish and shellfish from seafood from Cochin, Kerala, by Sanjeev and Stephen (1993) reported that 100% of the samples harbored *V. parahaemolyticus.* Later in a study in the same geographical area by Sudha et al. (2012), a prevalence of 45.1% was reported in finfish. *V. parahaemolyticus* was detected during all the months of sampling. Kerala is a tropical region and lies geographically close to the equator. In tropical regions the prevalence of *V. parahaemolyticus* is not correlated to temperature and its presence is found throughout the year (Deepanjali et al., 2005; Lekshmy et al., 2014). It is also reported that low salinity is not much detrimental to the growth of *V. parahaemolyticus* provided the presence of organic matter in the environment (Deepanjali et al., 2005). However, to avoid any bias due to decreased salinity, sampling was avoided during the monsoon months.

The virulent strains of *V. parahaemolyticus* are marked by their ability to produce β-hemolysis (Kanagawa phenomenon), a property conferred to them by the possession of either *tdh* gene or *trh* gene or both of these (Nishibuchi et al., 1989). In the present study, β-hemolytic *V. parahaemolyticus* was isolated from seafood and water samples. These water samples were used by fish vendors to sprinkle over the fish in the absence of ice. Thus the isolation of potentially pathogenic *V. parahaemolyticus* from these water samples suggests that these are possible sources of contamination. Previous studies carried out in Cochin, Kerala (India) reported KP positive *V. parahaemolyticus* in 20.5% of isolates from finfish and 22.9% of isolates from shellfish (Sanjeev and Stephen, 1995). Chakraborty et al. (2008) reported weak hemolysis in 10% of the *V. parahaemolyticus* isolates from seafood but did not find β-hemolysis in any of the isolates. Though the Kanagawa reaction is often used to identify the virulent strains, the chance to encounter such isolates from environmental samples are considerably less (Garrido et al., 2012). Abd-Elghany and Sallam (2013) reported that only 1.6% of the 120 samples tested gave positive Kanagawa reaction but 16% of the Kanagawa negative isolates were positive for the *tdh* gene.

The prevalence of *tdh*^+^ or *trh*^+^ strains in seafood and environment is reported to be less and constitutes 1–10% of the isolates, whereas most of the clinical *V. parahaemolyticus* isolates carry *tdh* and/or *trh* genes (Cook et al., 2002; Deepanjali et al., 2005; Fuenzalida et al., 2006; Letchumanan et al., 2015, 2014). Although there have been reports on the detection of *tdh* and *trh* gene from Cochin estuary and tiger shrimp culture environment in Cochin along the south-west coast, there are no major reports of detection of pathogenic *V. parahaemolyticus* carryin*g tdh* and *trh* virulence genes from seafood (Chakraborty and Surendran, 2009; Silvester et al., 2015). In India, isolation of pathogenic isolates of *V. parahaemolyticus* possessing *tdh* gene from retail seafood is rarely reported (Dileep et al., 2003; Pal and Das, 2010; Parthasarathy et al., 2016). This study demonstrated that potentially pathogenic isolates possessing the virulence genes are present in the seafood sold in retail markets of Cochin, Kerala. In the present study, the *tdh* gene amplification from certain seafood isolates produced faint amplification bands and certain non-specific amplifications. Gutierrez West et al. (2013) have previously reported faint amplification bands while using the Bej et al. (1999) primers, which required band purification and reamplification before sequencing. Similarly, in our study the sequences of two isolates that produced faint amplifications were confirmed as *tdh* gene. However, the clinical isolates used in this study displayed single prominent amplification bands. Thus the results of our study suggest that the presence of *tdh* positive *V. parahaemolyticus* in seafood or environmental samples is often undetected resulting in the underestimation of the actual levels. It may be either due to the presence of only single copy of *tdh* gene as compared to two in clinical strains or due to the mispriming or due to any other intrinsic factors. The conventionally employed multiplex PCR would be advantageous when testing the pathogenicity of the clinical isolates, but an elaborate protocol including testing the isolate for β-hemolytic activity, presence of *tdh*/*trh* gene in singleplex PCR amplifications using multiple primers and sequencing shall be used for environmental isolates. In the present study, KP positive isolates that are negative for the *tdh* and *trh* genes were detected. This is in consensus with the findings of Gutierrez West et al. (2013) where they detected similar isolates and suggested mechanisms that are not mediated by *tdh* and *trh* or the presence of variants of these genes that are not identifiable by the primers employed. Our study proves that the usual practice of relying only on virulence gene amplifications to detect the pathogenic strains of *V. parahaemolyticus* may be insufficient as many these isolates could be KP positive but *tdh* and *trh* negative. This should be read in conjunction with the reports of clinical strains negative for *tdh* and *trh* genes (Ronholm et al., 2016). Therefore, judging the *tdh* and *trh* negative isolates as non-hemolytic and non-pathogenic would be inappropriate. And hence, detection of β-hemolytic activity of the isolates is necessary to determine the pathogenicity of *V. parahaemolyticus*.

The prevalence of O1 serotype reported in our study is comparable with the results of Chao et al. (2009). The study on the characterization of *V. parahaemolyticus* from food, environmental, and clinical sources by DePaola et al. (2003) revealed that most of the food and environmental isolates belonged to O1 and O4 serotype. The serovariants of pandemic O3:K6 clone mostly belong to the O1 and O4 serotypes and thus the presence of these serotypes in the environment is significant (Nair et al., 2007). The results of our study revealed that there is no consensus among the results of three methods employed for the detection of pandemic isolates. Similar observations of variance in results among the methods used for detection of pandemic strains were made by Li et al. (2016) where the prevalence of GS-PCR, PGS PCR, Orf8, and HU-α positive isolates were reported to be 81.9, 38.9, 16.9, and 18.9%, respectively. Another study conducted in the eastern coastal region of India reported PGS-PCR and GS-PCR positive isolates to be, respectively, 6.5% and 2.4% of pathogenic isolates (Parthasarathy et al., 2016).

Recent trend indicates an increasing presence of MDR *V. parahaemolyticus* among the isolates from aquatic origin as compared to clinical samples (Xie et al., 2017). Isolation of multiple drug resistant strains of *V. parahaemolyticus* from fish landing centers and retail markets have been reported earlier from South India (Reyhanath and Kutty, 2014; Sudha et al., 2014). Though MDR *V. parahaemolyticus* were hardly encountered in our study, potentially pathogenic isolates resistant to second, third and fourth generation Cephalosporins were present in the seafood studied. Our study and the previous reports indicate that *V. parahaemolyticus* is increasingly becoming resistant toward third and fourth generation Cephalosporins (Vaseeharan et al., 2005; Saifedden et al., 2016; Yu et al., 2016; Lee et al., 2018; Park et al., 2018). The resistance toward ampicillin observed in *V. parahaemolyticus* isolates (79.3%) used in this study is comparable with the intrinsic resistance toward ampicillin shown by this bacterium elsewhere (Sudha et al., 2014; Chiou et al., 2015; Elmahdi et al., 2016; Yu et al., 2016). Among the three isolates that showed a MAR index of 0.2 or above one was positive for PGS-PCR. The multiple antibiotic resistance among the pathogenic isolates with pandemic potential could lead to untreatable illness and human health risk. The MAR index of 0.2 or above is significant and is considered “high-risk” source of contamination (Krumperman, 1983).

**FIGURE 4:**
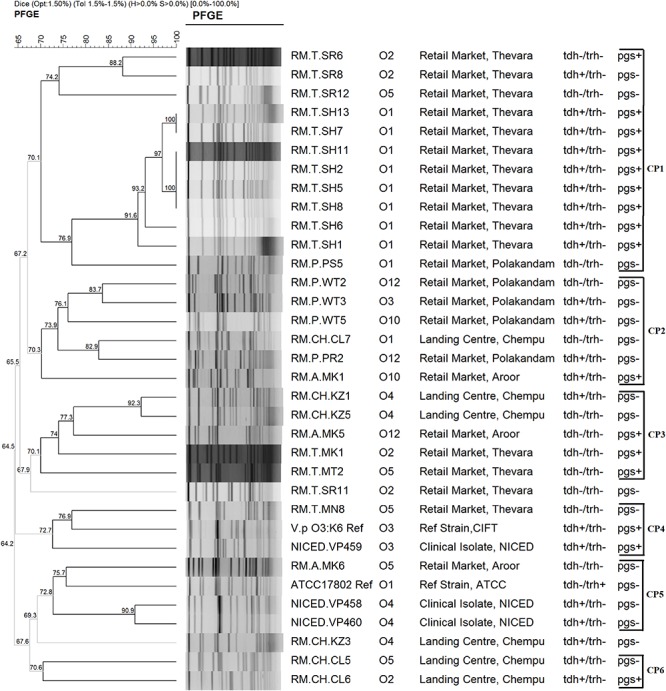
Dendrogram representing the cluster analysis using PFGE fingerprints.

The analysis of ERIC-PCR and PFGE fingerprints showed that there is geographical influence in clustering of the isolates in CE3 and CP1. All other clusters had isolates with different source, geographical location and virulence characteristics (Figures 3, 4). In a previous study on *V. parahaemolyticus* isolated from seafood from Cochin, Kerala using ERIC-PCR, grouping of isolates based on the source of isolation was reported (Chakraborty et al., 2013). Another study on genotyping of *V. parahaemolyticus* from the same geographical area reported that the isolates from different location and source fell into the same cluster (Silvester et al., 2017). Though our observations coincide with both these studies, the influence of geographical factor could be dismissed as all the isolates from single geographical location did not fall into one cluster. Two isolates from seafood (RM.A.MK6 and RM.P.PS5) that clustered along with the pandemic strains in ERIC-PCR analysis is understood to share genetic similarities with pandemic strains even though they were negative for pandemic specific PCR. These results are harmonious with that of PFGE where the isolate RM.A.MK6 was found to cluster along with the clinical isolates. ERIC-PCR and PFGE has been often used to study the genetic relatedness among the *V. parahaemolyticus* isolates from seafood, environmental and clinical samples (Wong et al., 1996; Wagley et al., 2008; Liu et al., 2009; Chen W. et al., 2012; Xie et al., 2017). Untypeablity of a high proportion of isolates due to degradation of DNA was reported to be a drawback for PFGE (Marshall et al., 1999; Wong and Lin, 2001; Chen W. et al., 2012). However, in our study, all the isolates were typeable by this technique. The analysis of ERIC-PCR and PFGE results suggest that at least three isolates (RM.T.MN8, RM.A.MK6, RM.P.PS5) have close genetic relationship with the pandemic and/or clinical isolates. Interestingly, all these isolates were negative for all the three pandemic specific PCR methods employed in this study. Therefore it could hardly be inferred that these isolates have any pandemic potential although it has genetic similarity with the clinical isolates. The pandemic serotypes like O4:K68, O1:K25, and O1:KUT (untypeable) have been reported to share similar AP-PCR profiles, ribotypes, and PFGE profiles with that of the O3:K6 serotype (Chowdhury et al., 2000; Nair et al., 2007). However, in this study, the PGS PCR positive isolates were not clustered with the pandemic strains both in ERIC-PCR as well as PFGE. Perhaps, this could be due to the difference in the genetic composition of environmental and clinical strains. To understand these aspects further research on various methods used in the identification of pandemic strains with both environmental and clinical isolates is essential.

Heterogeneity with respect to the distribution of O serotypes among the clusters was observed in ERIC fingerprint analysis. The analysis of PFGE patterns also showed isolates with multiple serotypes clustering together. All clusters had isolates belonging to different serotypes. This is in accordance with the observations of Wang et al. (2017) who reported that *V. parahaemolyticus* with different serotypes could share similar molecular types. In their study, no match was observed between serotype and PFGE cluster. MLST data have also confirmed that multiple serotypes occur in a single genetic lineage (Nair et al., 2007). It is apparent from these data that there is no correlation between genetic relatedness and serotype relatedness. The antibiotic profiles of the isolates characterized in this study did not coincide with the clustering of ERIC and PFGE. This is consistent with a previous study on antibiotic susceptibility of *V. parahaemolyticus* from ready-to-eat foods in China (Xie et al., 2016).

## Conclusion

This study confirmed the presence of potentially pathogenic *V. parahaemolyticus* in marine fish and shellfish sold in the retail markets in Cochin, Kerala along the south-western part of the Indian sub-continent. This is the first report of the isolation of *tdh* positive *V. parahaemolyticus* from seafood in Kerala (India). Our study suggest that the number of *tdh* positive *V. parahaemolyticus* in the environmental samples often goes undetected and a more elaborate protocol is required while dealing with environmental isolates. The clustering of three of the seafood isolates with the clinical strains in ERIC-PCR and PFGE shows that these isolates are genetically close to the clinical strain and might have an outbreak potential. The analysis of the antibiotic resistance pattern confirms the presence of potential pathogenic isolates of *V. parahaemolyticus* that exhibit resistance toward multiple antibiotics. The prevalence of pathogenic strains of *V. parahaemolyticus* in water and seafood samples and further, the presence of antibiotic-resistant strains and strains that are genetically related to the clinical strains is of public health significance. There is another health risk that may arise with cross contamination by such fish and shellfish to other samples of fish in the market. Many of these issues could be resolved by following strict hygiene at the point of sale, use of potable water to sprinkle over the fish and proper separation of the seafood products. Further to confront the possible implications of the presence of pathogenic *V. parahaemolyticus* in seafood, continuous surveillance of environmental and seafood samples and source tracking of clinical and environmental isolates is essential.

## Data Availability Statement

The datasets generated for this study can be found in the Genbank MK976027 and MK953547.

## Author Contributions

SN performed the experiments and wrote the manuscript. Supervision of experiments and validation of results was done by TJ. SP conducted the statistical analysis. MM performed review and editing. Conceptualization, guidance, and proofreading were done by KL.

## Conflict of Interest

The authors declare that the research was conducted in the absence of any commercial or financial relationships that could be construed as a potential conflict of interest.
