# Supplementary material for: Prevalence, Virulence Characterization, AMR Pattern and Genetic Relatedness of Vibrio parahaemolyticus Isolates From Retail Seafood of Kerala, India
Source: Front Microbiol. 2020 Apr 7;11:592. doi: 10.3389/fmicb.2020.00592 (PMC7154082; doi:10.3389/fmicb.2020.00592)
Supplement: Supplementary file 1 [file Table_1.docx]

Supplementary Material

# Supplementary Table S1: Isolate characteristics

|  | Isolate code | Source | Location | O serotype | KP | *toxR* | *tdh* | *trh* | GS PCR | PGS PCR |
| --- | --- | --- | --- | --- | --- | --- | --- | --- | --- | --- |
| 1 | RM.T.SR6 | Finfish | Retail market, Thevara | O2 | + | + | +f^*^ | - | - | + |
| 2 | RM.T.SR8 | Finfish | Retail market, Thevara | O2 | + | + | +f | - | - | - |
| 3 | RM.T.SR11 | Finfish | Retail market, Thevara | O2 | + | + | +f | - | - | - |
| 4 | RM.T.SR12 | Finfish | Retail market, Thevara | O5 | + | + | - | - | - | - |
| 5 | RM.T.SH1 | Shellfish | Retail market, Thevara | O1 | + | + | + | - | - | + |
| 6 | RM.T.SH2 | Shellfish | Retail market, Thevara | O1 | + | + | + | - | - | + |
| 7 | RM.T.SH5 | Shellfish | Retail market, Thevara | O1 | + | + | + | - | - | + |
| 8 | RM.T.SH6 | Shellfish | Retail market, Thevara | O1 | + | + | + | - | - | + |
| 9 | RM.T.SH7 | Shellfish | Retail market, Thevara | O1 | + | + | + | - | - | + |
| 10 | RM.T.SH8 | Shellfish | Retail market, Thevara | O1 | + | + | + | - | - | + |
| 11 | RM.T.SH11 | Shellfish | Retail market, Thevara | O1 | + | + | +f | - | - | + |
| 12 | RM.T.SH13 | Shellfish | Retail market, Thevara | O1 | + | + | + | - | - | + |
| 13 | RM.T.MK1 | Finfish | Retail market, Thevara | O2 | + | + | - | - | - | + |
| 14 | RM.T.MN8 | Finfish | Retail market, Thevara | O5 | + | + | - | - | - | - |
| 15 | RM.T.MT2 | Finfish | Retail market, Thevara | O5 | + | + | - | - | - | + |
| 16 | RM.CH.CL5 | Shellfish | Landing Centre, Chempu | O5 | + | + | +f | - | - | - |
| 17 | RM.CH.CL6 | Shellfish | Landing Centre, Chempu | O2 | + | + | +f | - | - | + |
| 18 | RM.CH.CL7 | Shellfish | Landing Centre, Chempu | O1 | + | + | +f | - | - | - |
| 19 | RM.CH.KZ1 | Finfish | Landing Centre, Chempu | O4 | + | + | - | - | - | - |
| 20 | RM.CH.KZ3 | Finfish | Landing Centre, Chempu | O4 | + | + | +f | - |  | - |
| 21 | RM.CH.KZ5 | Finfish | Landing Centre, Chempu | O4 | + | + | - | - | - | - |
| 22 | RM.A.MK1 | Finfish | Retail market, Aroor | O10 | + | + | +f | - | - | + |
| 23 | RM.A.MK5 | Finfish | Retail market, Aroor | O12 | + | + | - | - | - | + |
| 24 | RM.A.MK6 | Finfish | Retail market, Aroor | O5 | + | + | - | - | - | - |
| 25 | RM.P.PS5 | Finfish | Retail market, Polakandam | O1 | + | + | +f | - | - | - |
| 26 | RM.P.PR2 | Finfish | Retail market, Polakandam | O12 | + | + | +f | - | - | - |
| 27 | RM.P.WT2 | Water | Retail market, Polakandam | O12 | + | + | - | - | - | - |
| 28 | RM.P.WT3 | Water | Retail market, Polakandam | O3 | + | + | +f | - | - | - |
| 29 | RM.P.WT5 | Water | Retail market, Polakandam | O10 | + | + | +f | - | - | - |
| 30 | NICED.VP458 | Clinical source | Clinical isolate, NICED | O4 | + | + | +­ | - | - | - |
| 31 | NICED.VP459 | Clinical source | Clinical isolate, NICED | O3 | + | + | + | - | + | + |
| 32 | NICED.VP460 | Clinical source | Clinical Isolate, NICED | O4 | + | + | + | - | - | - |
| 33 | ATCC17802 | ATCC | Reference strain, ATCC | O1 | + | + | - | + | - | + |
| 34 | Vibrio parahaemolyticus O3:K6 | Clinical source | Reference strain, CIFT | O3 | + | + | + | - | + | + |

f*-faint amplification bands
